# Small interference RNA targeting tissue factor inhibits human lung adenocarcinoma growth in vitro and in vivo

**DOI:** 10.1186/1756-9966-30-63

**Published:** 2011-05-28

**Authors:** Chengcheng Xu, Qi Gui, Wenshu Chen, Leiming Wu, Wei Sun, Ni Zhang, Qinzi Xu, Jianing Wang, Xiangning Fu

**Affiliations:** 1 Department of General Thoracic Surgery, Tongji Hospital, Tongji Medical College, Huazhong University of Science and Technology, Wuhan, People's Republic of China; 2 Department of Oncology, Tongji Hospital, Tongji Medical College, Huazhong University of Science and Technology, Wuhan, People's Republic of China

**Keywords:** Lung adenocarcinoma, A549, Tissue factor, RNA interference, Gene therapy

## Abstract

**Background:**

The human coagulation trigger tissue factor (TF) is overexpressed in several types of cancer and involved in tumor growth, vascularization, and metastasis. To explore the role of TF in biological processes of lung adenocarcinoma, we used RNA interference (RNAi) technology to silence TF in a lung adenocarcinoma cell line A549 with high-level expression of TF and evaluate its antitumor effects in vitro and in vivo.

**Methods:**

The specific small interfering RNA (siRNA) designed for targeting human TF was transfected into A549 cells. The expression of TF was detected by reverse transcription-PCR and Western blot. Cell proliferation was measured by MTT and clonogenic assays. Cell apoptosis was assessed by flow cytometry. The metastatic potential of A549 cells was determined by wound healing, the mobility and Matrigel invasion assays. Expressions of PI3K/Akt, Erk1/2, VEGF and MMP-2/-9 in transfected cells were detected by Western blot. In vivo, the effect of TF-siRNA on the growth of A549 lung adenocarcinoma xenografts in nude mice was investigated.

**Results:**

TF -siRNA significantly reduced the expression of TF in the mRNA and protein levels. The down-regulation of TF in A549 cells resulted in the suppression of cell proliferation, invasion and metastasis and induced cell apoptosis in dose-dependent manner. Erk MAPK, PI3K/Akt pathways as well as VEGF and MMP-2/-9 expressions were inhibited in TF-siRNA transfected cells. Moreover, intratumoral injection of siRNA targeting TF suppressed the tumor growth of A549 cells in vivo model of lung adenocarcinoma.

**Conclusions:**

Down-regulation of TF using siRNA could provide a potential approach for gene therapy against lung adenocarcinoma, and the antitumor effects may be associated with inhibition of Erk MAPK, PI3K/Akt pathways.

## Background

Lung cancer is the leading cause of cancer-related death worldwide [1,2]. Lung adenocarcinoma, accounted for approximately 40% of all lung cancers, is currently one of the most common histological types and its incidence has gradually increased in recent years in many countries [3].

Tissue factor (TF), a 47-kDa transmembrane glycoprotein, primarily initiates the coagulation cascade by binding to activated factor VII (FVIIa) [4,5]. Under normal conditions, TF is highly expressed by cells which are not in contact with the blood, such as smooth muscle cells, mesenchymal and epithelial cells. In addition, numerous studies have reported that TF is aberrantly expressed in solid tumors, including cancers of the pancreas, prostate, breast, colon and lung [6,7], and TF can be detected on the surface of tumor cells and TF-bearing microparticles in the blood circulation shed from the cell surface [8,9]. The role of TF in coagulation has been much more focused on, and the association between tumor and coagulation was first revealed by Trousseau as long ago as 1865 [10]. Recently, the roles of TF in tumor growth, angiogenesis, and metastasis have become popular fields of research. Precious studies have been implicated that TF plays an important role in melanoma and pulmonary metastasis [11,12]. However, no study so far has demonstrated the antitumor effects and its antitumor mechanism via inhibition of TF expression by small interfering RNA (siRNA) in Lung adenocarcinoma. RNA interference (RNAi) is sequence-specific post-transcriptional gene-silencing process, which is initiated by double-stranded RNA (e.g. chemically synthetic small interfering RNAs) and then the RNA-induced silencing complex (RISC) degrades targeted mRNA and inhibits the protein expression [13]. Because of the effective, stable gene suppression by siRNAs, currently, RNAi technologies are widely used as knocking down genes in functional genomics [14].

In this study, we successfully used the RNA interference (RNAi) technology to silence the expression of TF in lung adenocarcinoma cell lines A549. In vitro and in vivo experiments described herein, we demonstrate that the capability of tumor growth and metastasis is reduced, and apoptosis is induced in TF-siRNA transfected A549 cells. In addition, Molecular mechanisms of the antitumor effects of TF knockdown are initially revealed, which could lay a foundation for genetic therapy for lung adenocarcinoma.

## Materials and methods

### Cell lines and cell culture

The human lung adenocarcinoma cell lines A549 was purchased from the Institute of Biochemistry and Cell Biology, Shanghai Institute for Biological Sciences, Chinese Academy of Sciences. Cells were grown in RPMI 1640 (Gibco) medium, supplemented with 10% fetal bovine serum (FBS), 100 U/ml penicillin and 100 ug/ml streptomycin in a humidified atmosphere of 5% CO2 at 37 °C. The cells in the logarithmic phase of growth were used in all experiments described below.

### Specific siRNAs and transfection

One siRNA oligonucleotides targeting human tissue factor (SiTF) [15] (accession no.M16553, the target mRNA sequences:5'-GCGCUUCAGGCACUACAAA-3'), one scrambled non-targeting siRNA (used for a negative control, Mock) and one fluorescent siRNA were designed and synthesized by Genepharma Co., Ltd (Shanghai, China). The sequences were as follows: SiTF, 5'-GCGCUUCAGGCACUACAAAtt-3' (sense) and 5'-UUUGUAGUGCCUGAAGCGCtt-3' (antisense); Mock, 5'-UUCUCCGAACGUGUCACGUtt-3' (sense) and 5'-ACGUGACACGUUCGGAGAAtt-3' (antisense). The 25 nM, 50 nM and 100 nM siRNAs were transfected into culture cells with Lipofectamine 2000 reagent (Invitrogen, Carlsbad, USA), according to the manufacturer's protocol. The cells were harvested 24, 48, or 72 h after transfection for analyses. Also as controls, A549 cells were either untreated or treated only with Lipofectamine 2000 reagent.

### Western blotting analysis

Cellular protein were extracted with RIPA lysis buffer and the concentrations were measured by the Bradford method using BCA Protein Assay Reagent [16]. Protein samples (20 ug/well) were separated by 10% SDS-PAGE, electrophoretically transferred to PVDF membranes, and the membranes were blocked, and then incubated with primary antibodies (1:2000) overnight at 4°C, followed by secondary antibodies against rabbit or mouse IgG conjugated to horseradish peroxidase (1:3000) for 2 hours at room temperature. Finally, after developed with ECL detection reagents, the protein bands of membranes were visualized by exposure to x-ray film. Protein expression was quantified by densitometry and normalized to β-actin expression. Anti-TF(sc-80952), anti-PI3K(sc-7174), anti-Akt(sc-9312)/phosphorylated Akt(sc-16646R), anti-Erk1/2(sc-93)/phosphorylated Erk1/2(sc-7383), anti-MMP-2(sc-10736)/-9(sc-12759), anti-VEGF(sc-507), and anti-β-actin(sc-130300) antibodies were obtained from Santa Cruz Biotechnology, Inc. (Santa Cruz, CA).

### Reverse Transcription-PCR

Total RNA was isolated from transfected cells with TRIzol reagent (Invitrogen, Carlsbad, CA) according to the manufacturer's protocol. Briefly, 1 ug total cellular RNA was reverse-transcribed by a First Strand cDNA Synthesis Kit (Amersham, Buckinghamshire, UK). Primers used for PCR amplification of TF were 5'-TGGAGACAAACCTCGGACAG-3' as the forward primer and 5'-ACGACCTGGTTACTCCTTGA-3' as the reverse primer, amplifying a 626bp fragment; and of GAPDH, the forward primer 5'-CCACCCATGGCAAATTCCATGGCA-3' and the reverse primer 5'-TCTAGACGGCAGGTCAGGTCCACC-3, amplifying a 600bp fragment. The following conditions were used for PCR: 94°C for 30s, 58°C for 30s, 72°C for 40s; 30 cycles and 72°C for 5 min for final extension. The PCR products were separated on 1% agarose gel, visualized under UV and photographed. The result was analyzed by Quantity One 4.6.2 software for the optical density.

### Cell proliferation assay

Cell proliferation was detected by MTT assay. A549 cells were seeded in 96-well plates at a density of 1 × 10^4 ^cells/well. After 24 h, the cells were transfected with siRNAs and cultured for 0-96 h. Cell proliferation was determined by adding MTT (5 mg/ml) and incubating the cells at 37°C further for 4 h, then the precipitate was solubilized by the addition of 150 ul/well DMSO (Sigma) and shaken for 10 min. Absorbance at a wavelength of 490 nm in each well was measured with a microplate reader (Bio-Tek ELX800, USA).

### Clonogenic assay

Cells transfected with siRNAs after 48 h were seeded in 6-well plates at a density of 600 cells/well and incubated for 2 weeks at 37°C in a humidified atmosphere of 5% CO2. The colonies were fixed with in 4% paraformaldehyde at room temperature for 20 min, stained with 0.1% crystal violet for 10 min, and finally, positive colony formation (more than 50 cells/colony) was counted and colony formation rate was calculated.

### Wound healing assay

A549 cells were transfected with siRNAs in 6-well plate. After 48 h, the cells were grown to confluence, and scratched with sterile P20 pipette tips. Plates were washed twice with PBS to remove detached cells and incubated with the complete growth medium without FBS. Cells migrated into the wounded area, and photographs were taken immediately (0 h) and 24 h, respectively. The result was expressed as a migration index: the area covered by the migrating cells (24 h)/ the wound area (0 h)

### Invasion and motility assay

Matrigel invasion assay was performed using Transwell chambers. Briefly, the 8-μm pore size filters were coated with 100 μl of 1 mg/ml Matrigel ((BD Biosciences, Bedford, MA). 500 ul RPMI1640 medium containing 10% FBS was added to the lower chambers. After transfection with siRNA for 48 h, Cells were harvested and homogeneous single cell suspensions (2 × 10^5 ^cells/ well) were added to the upper chambers. The invasion lasted for 24 h at 37°C in a CO2 incubator. After that, noninvasive Cells on the upper surface of the filters were carefully scraped off with a cotton swab, and cells migrated through the filters were fixed and stained with 0.1% crystal violet for 10 min at room temperature, and finally, examined and photographed by microscopy(×200). Quantification of migrated cells was performed. The procedure of motility assay was same to invasion assay as described above but filters without coating Matrigel.

### Flow cytometric analysis of apoptosis

After transfection for 48 h, cells in 6 well plates were harvested in 500 ul of binding buffer, stained with 5 ul AnnexinV-FITC and 5 ul propidium iodide for 10 min using a apoptosis Kit(keyGen, Nanjing, China), and subjected to flow cytometric analysis by a CycleTEST™ PLUS (Becton Dickinson, San Jose, CA) within 1 h. The results were quantitated using CellQuest and ModFit analysis software.

### Nude mouse xenograft model

Female BALB/c nu/nu mice (4-5 weeks old) were purchased from Nanjing Qingzilan Technology Co., Ltd (Nanjing, China). Animal treatment and care were in accordance with institutional guidelines. A549 cells(1 × 10^7^) were suspended in 100 ul PBS and injected subcutaneously in the right flank region of nude mice. After 2 weeks, when the tumor volume reached 50-100 mm^3^, mice were randomly divided into three groups (5 mice per group): (1) control group, untreated; (2) mock group, intratumoral injection of 50 ug scramble siRNA every 5 days; (3) SiTF group, intratumoral injection of 50 ug TF-siRNA every 5 days [17-19]. The tumor diameters were measured 2 times a week with a caliper. The tumor volume (mm^3^) was calculated according to the following formula: length × width^2^/2 [17,18]. All mice were sacrificed humanely after 5 times of treatment, and the resected tumors were weighed.

### Statistical analysis

All data were shown as mean ± standard deviation (SD). Statistical significance was determined by analysis of variance (ANOVA) using SPSS 12.0 software package. The level for statistical differences was set at P < 0.05.

## Results

### Knockdown of TF expression by TF-siRNA in NSCLC cell lines A549

**Figure 1:**
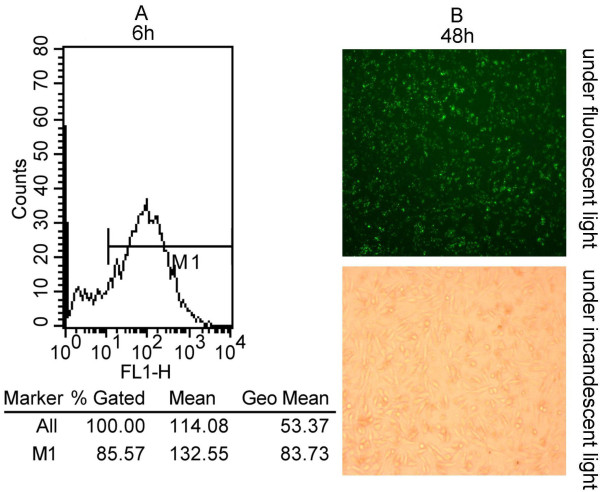
**Efficient delivery of siRNA into lung adenocarcinoma cells**. (A): Detection of transfection efficiency by flow cytometry. Transfection efficiency was maintained at over 85% for 6 h post-transfection. (B): Detection of transfection efficiency by fluorescence microscopy. High efficiency of transfection with fluorescent siRNA (green) in A549 cells were easily identified for 48 h post-transfection (×100).

**Figure 2:**
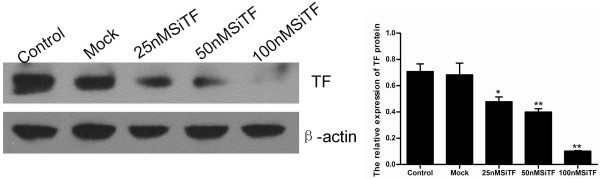
**TF-siRNA suppressed the TF protein expression in lung adenocarcinoma cells**. 48 h after transfection, the concentration of 100 nM TF-siRNA (100 nM SiTF group) was identified as the most efficient to knock down the expression of TF by Western blot. *P < 0.05, **P < 0.01 versus mock.

**Figure 3:**
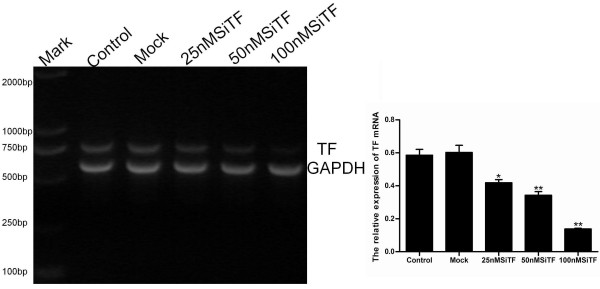
**TF-siRNA suppressed the mRNA expression in lung adenocarcinoma cells**. The concentration of 100 nM TF-siRNA (100 nM SiTF group) was identified as the most efficient to knock down the expression of TF by RT-PCR. *P < 0.05, **P < 0.01 versus mock.

To make sure the transfection efficiency of siRNA in A549 cells, uptake of fluorescently labeled scrambled siRNAs (25 nM, 50 nM and 100 nM) was detected by flow cytometry and fluorescence microscopy after 6 h and 48 h post-transfection. It showed a high-efficiency transfection that more than 85% cells displayed green fluorescence with 100 nM fluorescent siRNA (Figure 1). When cells were treated with TF-targeting siRNA (25 nM, 50 nM and 100 nM SiTF) and the scramble siRNA (Mock, 100 nM) for 48 h, the mRNA and protein expressions of TF were examined by RT- PCR and Western blot. As shown in Figure 2 and Figure 3, the Mock did not affect the expression levels of TF, but in 25 nM, 50 nM and 100 nM SiTF groups, compared with mock, the TF expression decreased at both protein and mRNA levels. Specially, 100 nM SiTF indicated a 80-85% reduction of TF expression in A549 cells. These results demonstrated that the TF-targeting siRNA was efficient to knock down the expression of TF in A549 cells.

### Inhibition of cell proliferation and colony formation by TF-siRNA

**Figure 4:**
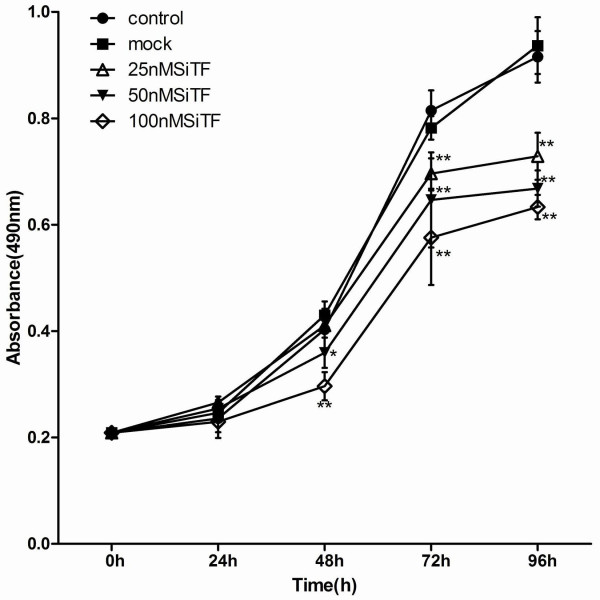
**Knockdown of TF with TF-siRNA inhibited cell proliferation of lung adenocarcinoma cells in vitro**. TF-siRNAs transfected A549 cell growth was significantly attenuated in a time- and dose-dependent manner compared with mock. *P < 0.05, **P < 0.01 versus mock.

**Figure 5:**
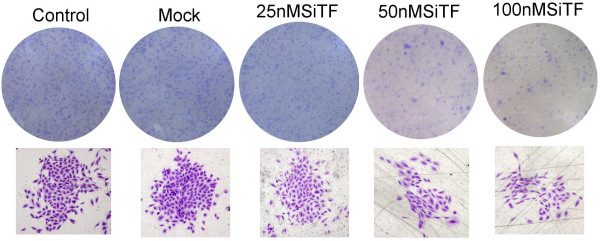
**Knockdown of TF with TF-siRNA inhibited colony formation of lung adenocarcinoma cells in vitro**. Representative images of the colony formation assay were shown.

**Figure 6:**
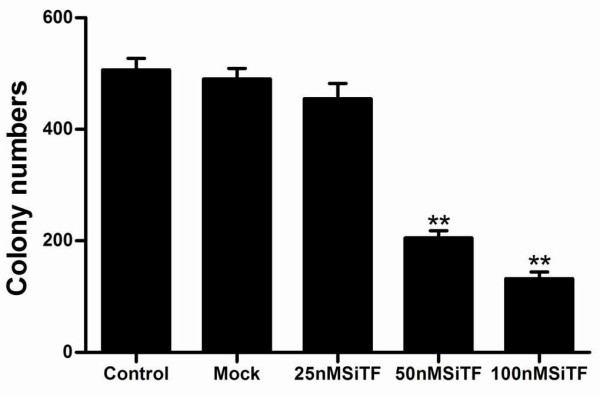
**Bar graph of the colony formation assay**. The result demonstrated that high concentrations of 50 nM and 100 nM TF-siRNA significantly attenuated the colony formation rate of lung adenocarcinoma cells. **P < 0.01 versus mock.

Since previous studies have shown that the expression of TF associated with tumor growth [20-22], the effect of TF siRNA on lung adenocarcinoma cell proliferation was determined by MTT assay. As shown in Figure 4, after 24 h-96 h transfection of TF siRNA into A549 cells, cell proliferation was remarkably inhibited in a time- and dose-dependent manner, when compared with control and mock groups. Inhibition of cell proliferation at 50 nM and100 nM began at 48 h post-transfection, but at 25 nM was observed at 72 h post-transfection, and higher concentrations of TF siRNA had greater effects. In addition, the colony formation assay further revealed effects of TF knockdown on growth properties of A549 cells. 50 nM and100 nM SiTF groups, but not 25 nM SiTF group had lower positive colony formation than control and mock groups, and it also seemed to depend on doses (Figure 5 and Figure 6). Overall, down-regulation of TF by siRNA resulted in a negative effect on growth of lung adenocarcinoma cells.

### Attenuation of the migration/invasion ability by TF-siRNA

**Figure 7:**
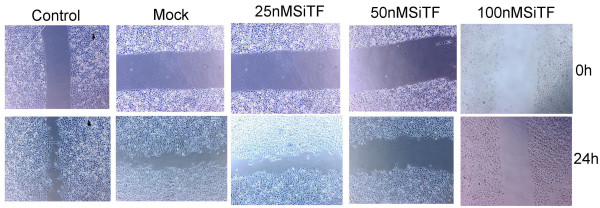
**Knockdown of TF with TF-siRNA attenuated the migration ability of lung adenocarcinoma cells in vitro**. Representative images of the wound healing assay were shown (×40).

**Figure 8:**
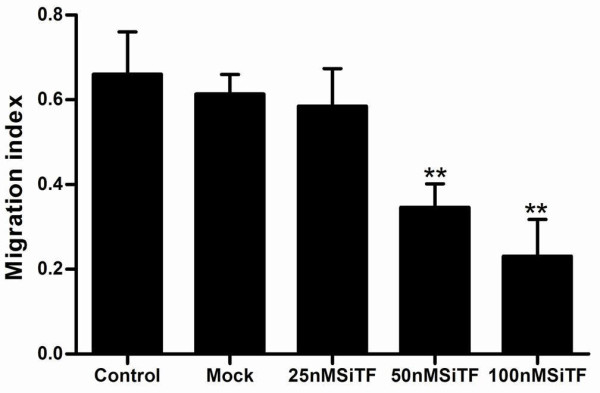
**Bar graph of the wound healing assay**. Bar shows the means percentage of wound area covered by migrating A549 cells. A549 cells treated with 50 nM and 100 nM TF-siRNA remarkably decreased the cell motility. **P < 0.01 versus mock.

**Figure 9:**
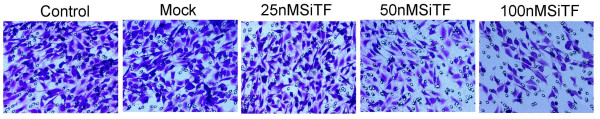
**Knockdown of TF with TF-siRNA attenuated the migration ability of lung adenocarcinoma cells in vitro**. Representative images of the mobility assay were shown (×200).

**Figure 10:**
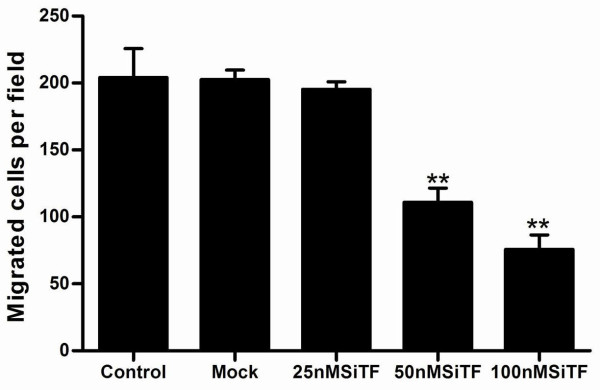
**Bar graph of the mobility assay**. Bar represents the mean number of the cells per field. Silencing TF by 50 nM and 100 nM TF-siRNA inhibited cell migration in lung adenocarcinoma cells. **P < 0.01 versus mock.

**Figure 11:**
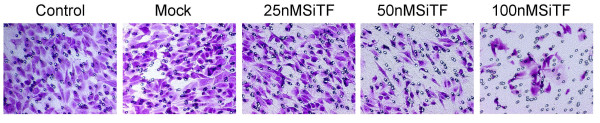
**Knockdown of TF with TF-siRNA attenuated the invasion ability of lung adenocarcinoma cells in vitro**. Representative microscopy images of the invasion assay are shown(×200).

**Figure 12:**
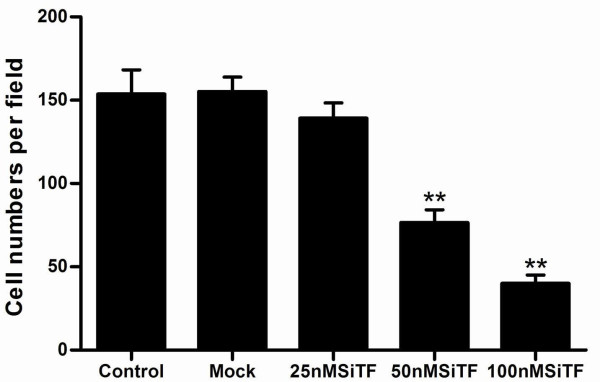
**Bar graph of the invasion assay**. Bar represents the mean number of the cells per field. The invasion assay was consistent with the migration assay and showed that the high concentration of 50 nM and 100 nM TF-siRNA attenuated the invasion ability of lung adenocarcinoma cells. **P < 0.01 versus mock.

Tumor cell migration and invasion are two critical steps in cancer metastatic process [23]. To verify the effect of TF-siRNA on the migration ability, A549 cells were tested by wound healing assay and the mobility assay. Figure 7 and Figure 8 show that the cells in 50 nM and 100 nM SiTF groups demonstrated an attenuated capacity of impaired migration, when compared to control and mock groups. Moreover, untreated and transfected cells were seeded on transwell chambers with uncoated filters. After incubation for 24 h, the motility potential of transfected cells at 50 nM and 100 nM TF-siRNA was significantly suppressed (Figure 9 and Figure 10). In addition, the invasion assay using Matrigel-coated Transwell chambers showed that 50 nM and 100 nM TF-siRNA transfected cells that passed through the Matrigel-coated membranes were much more than parental cells and the cells transfected with scrambled siRNA, and it indicated that the invasive capacity was markedly decreased (Figure 11 and Figure 12). These results suggested that TF-siRNA attenuated the metastatic potential of lung adenocarcinoma cells in vitro.

### Promoted apoptosis in A549 cells by TF-siRNA

**Figure 13:**
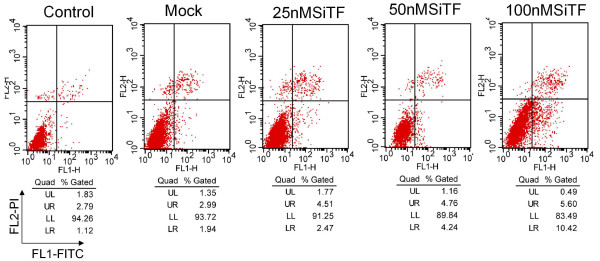
**Knockdown of TF with TF-siRNA induced apoptosis of lung adenocarcinoma cells**. The transfected cells, labeled with AnnexinV-FITC and propidium iodide, were subjected to flow cytometric analysis. Two parameter histogram Dot Plot displayed FL1-FITC on the x axis and FL2-PI on the y axis. The result showed that TF-siRNA increased the apoptotic rate in A549 cells in a dose-dependent manner.

To evaluate further whether knockdown of TF induces A549 cells apoptosis, at 48 h after transfection, the cells were harvested and analyzed by flow cytometry. As shown in Figure 13, the apoptosis rates of 25 nM, 50 nM and 100 nM SiTF groups were 7.0%, 9.0% and 16.0%, respectively, which were higher than 4.0% in control and 4.8% in mock groups, and indicated a dose-dependent increase.

### Molecular mechanisms of the antitumor effects by TF-siRNA

**Figure 14:**
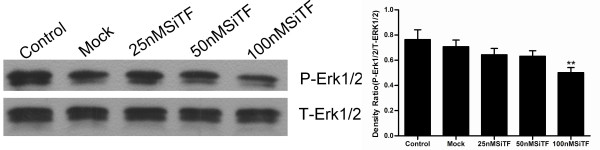
**Western blot analysis of Erk1/2 by silencing TF by siRNA in lung adenocacinoma cells in vitro**. Representative images were shown and bar represented that the protein relative expression levels of phosphorylated Erk1/2 (P-Erk1/2) in 100 nM SiTF group were decreased. **P < 0.01 versus mock.

**Figure 15:**
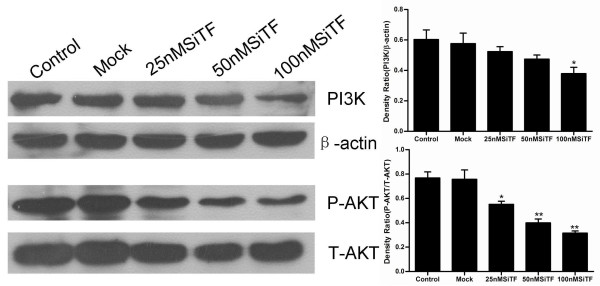
**Western blot analysis of PI3K/Akt by silencing TF by siRNA in lung adenocacinoma cells in vitro**. Representative images were shown and bar represented that the protein relative expression levels of PI3K in 100 nM SiTF group and phosphorylated Akt (P-AKT) in 25 nM, 50 nM and 100 nM SiTF groups were decreased. *P < 0.05, **P < 0.01 versus mock.

**Figure 16:**
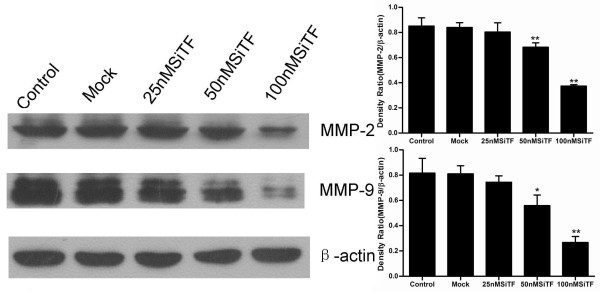
**Western blot analysis of MMP-9/-2 by silencing TF by siRNA in lung adenocacinoma cells in vitro**. Representative images were shown and bar represented that transfection with 50 nM and 100 nM TF-siRNA suppressed the MMP-9/-2 expression. *P < 0.05, **P < 0.01 versus mock.

**Figure 17:**
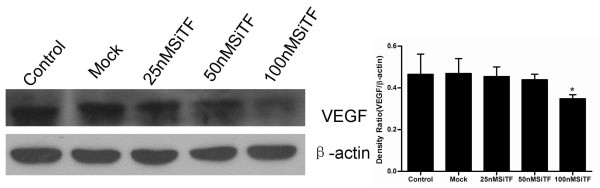
**Western blot analysis of VEGF by silencing TF by siRNA in lung adenocacinoma cells in vitro**. Representative images were shown and bar represented that the protein expression of VEGF of 100 nM SiTF group was decreased. *P < 0.05, **P < 0.01 versus mock.

The protein from transfected cells was extracted to examine the effects of TF-siRNA on some important cytokines and signaling molecules. After 48 h of transfection, the protein relative expression levels of phosphorylated Erk1/2 and PI3K in 100 nM SiTF group and phosphorylated Akt in 25 nM, 50 nM and 100 nM SiTF groups were decreased, while that in control and mock groups had no differences (Figure 14 and Figure 15). Furthermore, compared to control and mock groups, transfection with high concentrations of 50 nM and 100 nM TF-siRNA suppressed the MMP-9/-2 expression (Figure 16), and the protein expression of VEGF of 100 nM SiTF group was decreased (Figure 17). These data demonstrated that knockdown of TF by siRNA may inhibit Erk1/2 MAPK, PI3K/Akt signaling pathway, MMP-9/-2 and VEGF, which all play an important role in tumor progress.

### Inhibition of tumor growth of lung adenocarcinoma cells in nude mice by TF-siRNA

**Figure 18:**
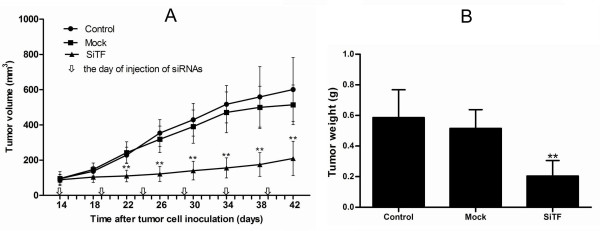
**Tumor volume curve and bar graph of tumor weight on the 42nd day when mice were killed**. (A): The curve showed that the tumor growth of SiTF group from days 22 to the end was significantly inhibited compared to that of control and mock groups. (B): Bar represented that the tumor weight of SiTF group was decreased than that of control and mock group. **P < 0.01 versus mock.

**Figure 19:**
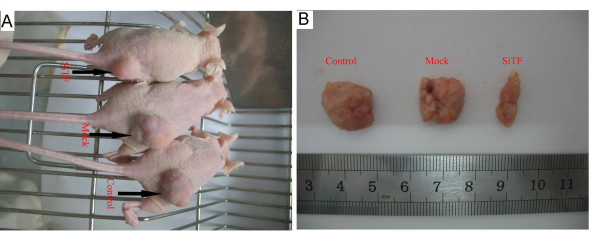
**Knockdown of TF by siRNA inhibited the tumor growth of lung adenocarcinoma cells in nude mice**. (A and B): Representative images showed that the tumor size of SiTF group was markedly smaller on the 42nd day after tumor cells inoculation than that of control and mock group.

**Figure 20:**
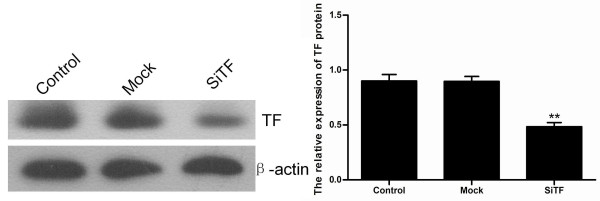
**TF-siRNA inhibited the protein expression of TF in vivo as determined by Western blot**. Representative images were shown and bar represented that the relative expression of TF in SiTF group was significantly inhibited compared to that in control and mock groups. **P < 0.01 versus mock.

Intratumoral injection with TF-siRNA was performed to investigate whether TF-siRNA had the effect of inhibition on tumor growth in vivo. A nude-mouse model of human lung adenocarcinoma xenograft was established, and when the tumor volume reached 50-100 mm^3^, intratumoral treatment with TF-siRNAs was started and repeated every 5 days for a total of 5 times. As shown in Figure 18A, the tumor volume of SiTF group from days 22 to the end was significantly smaller than control and mock groups, whereas there was no statistical difference between control group and mock group during the experiment. All mice were sacrificed on the 42nd day, and the final tumor volume and weight in SiTF group (209.6 ± 97.6 mm^3 ^and 0.21 ± 0.10 g, n = 5) were markedly smaller than that in control group (600.8 ± 182.0 mm^3 ^and 0.59 ± 0.18 g, n = 5) and mock group (513.8 ± 112.6 mm^3 ^and 0.52 ± 0.12 g, n = 5) (Figure 18 and Figure 19). In addition, the relative protein expression of TF in SiTF group was decreased significantly, but there was no statistical significance between control group and mock group (Figure 20). After all, these results indicated that intratumoral injection with TF-siRNA suppressed the tumor growth of lung adenocarcinoma cells in vivo.

## Discussion

Despite advances in the medical and surgical treatments, lung cancer is the leading cause of cancer deaths [1]and because of intrinsic properties of lung adenocarcinoma which cells show a high ability to rapid progress, it has a poor prognosis in main histological types of lung cancer [24,25]. Tumor progression includes tumor cell proliferation, invasion (loss of cell to cell adhesion, increased cell motility and basement membrane degradation), vascular intravasation and extravasation, establishment of a metastatic niche, and angiogenesis [23,26,27]. Therefore, how to effectively inhibit the proliferative and metastatic biological behavior of Lung adenocarcinoma cells is a key problem to improve the outcome.

Recent studies have implicated that TF plays an important role in biological processes of many cancers, and the main mechanism is mediated via angiogenesis [28,29]. In non-small-cell lung carcinomas, the increased TF expression associated with high VEGF levels and microvessel density has gained widespread acceptance [6,30]. However, A definite conclusion that silencing the expression of TF in lung adenocarcinoma affects the tumor cell proliferation, apoptosis and prometastatic processes such as migration and matrix degradation have not yet been established.

In this study, we have shown that chemically synthesized siRNAs specifically targeting TF successfully knocked down the expression of TF in both protein and mRNA levels by 80% to 85% in human lung adenocarcinoma cells A549. Then the assays as described above detected the effects on biological behavior of A549 cells in vitro. By the MTT and clonogenic assays, we were able to first show that the proliferation of the TF-siRNA transfected lung adenocarcinoma cells is significantly inhibited in vitro, but previous studies have failed to show that in colorectal cancer cells and B16F10 melanoma cells [11,12,31]. Using wound healing and transwell assays, TF-siRNA attenuated the potential of invasion and metastasis in lung adenocarcinoma cells. Furthermore, flow cytometric analysis revealed that knockdown of TF expression induced apoptosis in A549 cells. According to these results, we believed that besides participating in angiogenesis, TF also plays a key role in cell proliferation and metastasis of lung adenocarcinoma.

After binding of FVIIa, the TF forms a high affinity complex with FVIIa or FVIIa-FXa, and other than initiating the coagulation cascade, the complex induce signal transduction by binding to a family of transmembrane domain G protein-coupled cell surface receptors called protease-activated receptors (PARs), specially, PAR-1/-2 [32], which are expressed by numerous tumor cells and tissues [33,34]. In the tumor, it has recently emerged as important players in growth and metastasis, but previous studies have lacked information about the downstream signal pathways induced by the inhibition of the TF expression via TF-siRNA in lung cancer cells. In the current study, we established that down-regulation of TF expression in lung adenocarcinoma cells suppressed the Erk1/2 MAPK and PI3K/Akt signal pathways, which are well recognized for mediating cell proliferation and apoptosis [35,36]. Therefore, the result explains, at least in part, why TF-siRNA inhibited the cell proliferation and induced the apoptosis in A549 cells. Furthermore, the expressions of MMP-2/-9 also were down-regulated in TF-siRNA transfected cells, and it may suggest that MMP-2/-9 are the downstream products of the TF complex induced cell signaling. MMPs are a family of enzymes that degrade proteins in tissue extracellular matrices, which are clearly involved in cancer progression, including tumor cell degradation of basement membranes and stroma and blood vessel penetration [27]. Consequently, the reduction of MMP-2/-9 by TF-siRNA exactly results in attenuating the metastatic potency of lung adenocarcinoma cells.

Besides experiments in vitro that give new insights into the antitumor effects of TF-siRNA in lung adenocarcinoma, we used a nude mouse xenograft model of lung adenocarcinoma to better evaluate the TF-siRNA effects in vivo. Since in vitro results indicated that knockdown of TF by chemically synthesized siRNA lasted for about 5 days, the mice received intratumoral injection of TF-siRNA every 5 days of total 5 times to down-regulate the expression of TF. Through monitoring the tumor volume for about 4 weeks after injection, we found that the tumor growth in the treated mice with TF-siRNA was strongly suppressed. The results were in agreement with the nude mice bearing tumors of human breast cancer (MDA-MB-231) treated with EF24 conjugated to FVIIa [37]. Combined these findings in vitro and vivo, we confirmed the close relationship between TF and tumor growth, vascularization, and metastasis in lung adenocarcinoma.

## Conclusions

In summary, our findings clearly demonstrate that TF plays a crucial role in lung adenocarcinoma tumor growth and metastasis. This shows the first study in which silence of TF expression in lung adenocarcinoma cells by TF-siRNA could inhibit tumor growth and metastasis in vitro and in vivo, and the antitumor effects may be associated with inhibition of Erk MAPK, PI3K/Akt signal pathways in lung cancer. Therefore, RNA interference targeting TF may be a useful potential tool for the gene therapy of lung adenocarcinoma, and even other cancers at high level of TF expression.

## Abbreviations

ERK: extracellular signal-regulated kinase; MAPK: mitogen-activated protein kinase; MMP: matrix metalloproteinase; PARs: protease-activated receptors; PI3K: phosphoinositide 3-kinase; RNAi: RNA interference; siRNA: small interfering RNA; TF: tissue factor; VEGF: vascular endothelial growth factor.

## Competing interests

The authors declare that they have no competing interests.

## Authors' contributions

XC and GQ have contributed to the research design, the data collection and manuscript writing. CW, WL, SW, ZN, XQ and WJ have contributed to manuscript writing. FN has contributed to the research design and manuscript writing. All authors read and approved the final manuscript.
